# The association between nutritional status and functional limitations among centenarians: a cross-sectional study

**DOI:** 10.1186/s12877-021-02312-9

**Published:** 2021-06-21

**Authors:** Yang Song, Miao Liu, Wang-ping Jia, Ke Han, Sheng-shu Wang, Yao He

**Affiliations:** 1 Institute of geriatrics, the 2nd Medical Center, State Key Laboratory of Kidney Disease, Beijing Key Laboratory of Aging and Geriatrics, National Clinical Research Center for Geriatrics Diseases, Chinese PLA General Hospital, 28 Fuxing Road, 100853 Beijing, China; 2 Graduate school of Chinese PLA General Hospital, 28 Fuxing Road, 100853 Beijing, China

**Keywords:** Centenarians, Malnutrition, Functional limitations, ADL, MNA-SF

## Abstract

**Background:**

Although there have been studies on the association between nutritional status and functional limitations, there were few studies on Asian centenarians in community. Therefore, this study aims to identify associations between nutritional status and functional limitations among centenarians in China.

**Methods:**

This cross-sectional study was conducted with the data from the China Hainan Centenarian Cohort Study. These data ultimately included basic characteristics, hematologic indicators, and chronic disease status for 1,002 centenarians. The nutritional status was evaluated using the Mini Nutritional Assessment Short-Form scale. The functional limitations were assessed using the activities of daily living (ADL) scale, namely Barthel Index and Lawton Scale. The association between nutritional status and ADL was assessed using multivariate logistic regression models.

**Results:**

In this study, the prevalence of malnutrition was 20.8 % among centenarians, basic ADL (BADL) limitation was 28.6 %, and instrumental ADL (IADL) limitation was 64.7 %. As the nutritional status deteriorated, the risk of ADL limitations increased in total population (BADL limitation: OR = 17.060, 95 % CI: 8.093-35.964; IADL limitation: OR = 11.221, 95 % CI: 5.853-21.511; *p* for trend < 0.001). Similar results were found in both men and women after stratifying sex but were more prominent in women.

**Conclusions:**

Malnutrition is associated with functional limitations among centenarians in China and more pronounced among women.

## Background

The global older population is increasing; this makes us facing the aging population and the health problems of the older. Because in the aging process, the human body inevitably experiences various functional declines; the problem of functional limitations [1] and deterioration of nutritional status [2] are more prominent.

Aging is a complex process representing a myriad of overlapping sets of influences and changes that accrue over time [3]. With the decline of muscle strength and physical function during aging, the older will face a high risk of physical function limitations [4]. Existing studies on function limitations caused by aging have also found that unhealthy lifestyles (e.g., lack of physical activity) and chronic diseases (e.g., diabetes, hypertension, and heart disease) are related to function limitations in later life activities [5, 6].

Malnutrition is associated with many adverse events in the aging process of the older [7], which heavily affect health status and life quality [8]. Especially in terms of function, it has been proved that malnutrition can lead to functional limitations in the older [9–11]. This is because nutritional status may trigger changes in the structure and function of muscles. There are functional limitations and physical disability in older caused by muscle loss and other reasons [12].

However, the research on the relationship between nutritional status and functional limitations of centenarians is limited. Current research mainly focuses on European populations and the sick older [13–16], with a lack of studies on Asian populations and community older. Centenarians, as the “template population” of population aging, have reference value for the extension of their life span and health to the general older population [17]. The longevity of centenarians in China’s Hainan Province is closer to natural longevity than that of those in areas with higher level of economic and medical. This provides a unique sample for the study of nutritional and functional limitations of the natural longevity population. Therefore, this study aims to identify the association between nutritional status and functional limitations among Chinese centenarians in community, and to supplement the evidence for this association in the Asian centenarian population.

## Methods

### Study design and participants

We performed a cross-sectional analysis with the data of centenarians-based from the China Hainan Centenarian Cohort Study (CHCCS) from 2014 to 2016. The study sample of CHCCS was drawn from Hainan province, China through a full sample survey process [18]. The following inclusion criteria were used to recruit participants: (1) was 100 years or older by 1 June 2014; (2) volunteered to participate in the study and provided written informed consent; and (3) can get in touch with centenarians. Briefly, CHCCS is a study aiming to investigate health determinants and aspects of the centenarian population living in Hainan, China.

In 2014, at the time of the baseline survey, the older aged 100 years or over were considered eligible. Examining the ID numbers and on-site confirmations of all participants led to the choice of 1,473 centenarians as the target sample. Finally, centenarians who were unable to participate in all surveys were excluded, including 268 older who died before the interview, 124 older who unable to participate in the physical examination, and 79 older who declined to participate. 1,002 centenarians (180 men and 822 women) were included in our survey. These centenarians were not living in institutions, such as nursing homes. They live either with their families or alone in the community. As of the end of the survey, all the 1002 centenarians were alive.

### Data collection and definitions

In this survey, nurses familiar with the situation and language of the survey area completed questionnaires survey, physical examinations, and laboratory tests. In rare participants (e.g., centenarians with cognitive impairment), relatives of the centenarian were interviewed. Data were collected and sorted by two fully trained staff using the same equipment and software.

Basic characteristics included age, sex, educational level, residence type, physical activity, BMI, hematologic indicators, and chronic diseases. Age was ascertained from national ID cards. Educational level was categorized into “primary school and below” and “junior high school and above”. The residence type was categorized into “living alone” and “living with family”. The physical activity was assessed by answering “how many times did you do physical activities related to independent life per week”. We defined doing physical activities more than once a week as “physical activity”. Otherwise, it is “lack of physical activity”.

Body Mass Index (BMI) was calculated by weight and height. Weight and height were measured on a horizontal platform scale and a wall-mounted stadiometer to the nearest and 0.1 kg and 0.1 cm, respectively. Each parameter was measured twice, and the reported results were the averages of these duplicate measurements. Samples of venous blood were collected and transported by a standardized process, which analyzed by an automatic biochemical analyzer (COBAS c702; Roche Products Ltd., Basel, Switzerland) in the Hainan Branch of the Chinese People’s Liberation Army General Hospital. The hematologic indicators included hemoglobin (Hb), serum albumin (ALB), and total cholesterol (TC).

Participants were asked whether they had any current chronic diseases including diabetes, hypertension, and heart disease. Chronic diseases were identified based on self-reports combined with the medical records of the participants. For example, diabetes was defined as fasting blood glucose ≥ 7.0 mmol/L, or postprandial blood glucose ≥ 11.1 mmol/L, or currently using hypoglycemic drugs.

### Functional limitations

The functional limitations were assessed using the activities of daily living (ADL) scale [19], which was usually used to estimate the decline or even loss of body function among older. Activities of daily living are basic activities necessary for people to take care of themselves, maintain personal hygiene, and carry out independent social activities in daily life [20]. The Barthel index and Lawton scale evaluated basic ADLs (BADLs) and instrumental ADLs (IADLs), respectively; their validity has been well established in Chinese older [21, 22] and centenarians [23]. The more scores centenarians obtained in Barthel index and Lawton scale, the better their ADL. In this study, we used face-to-face questionnaires to assess centenarians’ ADL and conduct on-site inspections to ensure effectiveness and reliability.

The Barthel index includes the fundamental skills typically needed to manage basic physical needs, comprised of various functional skills in different areas [24]. It includes ten items including bowel control, bladder control, grooming, bathing, toilet use, dressing, feeding, stair climbing, transferring from bed to chair, and mobility [19]. The final score ranges from 0 (full dependence) to 100 (full independence), in 5-point intervals. Lawton scale is an appropriate tool for assessing independent living skills, which includes using the telephone, food preparation, housekeeping, laundry, handling finances, shopping, mode of transportation, and using own medications [25]. The final score ranges from 0 (full dependence) to 8 (full independence), in 1-point intervals.

As a special group, the activities of centenarians may be different from other age clusters. Quantifying their ADL using the same score would be unfair. Therefore, binary variables of ADL/IADL were constructed, which were “BADL limitation” and “No BADL limitation” / “IADL limitation” and “No IADL limitation. We define “BADL limitation” as ≤ 60 points [26], and “IADL limitation” as ≤ 2 points [13].

### Nutritional status

Nutritional status was assessed by the Mini Nutritional Assessment Short-Form (MNA-SF). It has been verified and widely accepted malnutrition risk screening in the older, with good sensitivity and high correlation [27]. The total weighted MNA-SF scores range from 0 to 14, with 12 to 14 indicating normal nutrition, 8 to 11 indicating being at the risk of malnutrition, and ≤ 7 indicating malnutrition. In this study, they were represented by “normal”, “at-risk”, and “malnutrition”.

### Statistical analyses

Descriptive statistics were performed using mean ± standard deviation for normally distributed continuous variables, and median (M) ± interquartile range (IQR) for non-normally distributed continuous variables. Categorical variables were expressed as n (%). Statistical differences were compared using independent samples T-test for continuos variables and Chi-square test for categorical variables. Logistic regression models were employed to estimate the odds ratios (ORs) and 95 % confidence interval (CI) of risk factors associated with functional limitations. Meanwhile, this binary coding of ADL/IADL variables was also used as dependent variables in the multivariable regression analysis, and BMI was excluded from the model because of multicollinearity with MNA-SF score. Last, according to different population, we divided them into three groups: total group, man group and woman group. And considered three types of variables: basic characteristics (age, sex, educational level, residence type, physical activity), hematological indicators (Hb, ALB, TC), and chronic diseases (diabetes, hypertension, heart disease). Run a logistic regression model (crude model) that includes only covariates of nutritional status, following by entering basic characteristic variables (model 1), hematologic indicators variables (model 2), and chronic diseases variables (model 3). Statistical significance was set at 0.05 (two sides). IBM SPSS Statistics for Windows, version 19.0 (IBMCorp., Armonk, N.Y., USA) and statistical software packages R (http://www.R-project.org, The R Foundation) were utilized for statistical data analysis.

## Results

Table 1 summarizes the basic characteristics of centenarians in different nutritional status. The study participant was on average 102 years old with 82.0 % women. Most centenarians had primary education or below (98.0 %), live with their families (86.1 %), and lack of activity (87.1 %). As the nutritional status declined, hematologic indicators also declined. Participants had a higher prevalence of hypertension, and a lower prevalence of diabetes and heart disease. There was no statistical difference in the prevalence of diabetes, hypertension, and heart disease under different nutritional status.

**Table 1:** Distribution of baseline characteristics of centenarians in different nutritional status

Characteristics	Total (*n* = 1002)	Nutritional status			*p*-Value ^a^
		**Normal (*n* = 123)**	**At-risk (*n* = 671)**	**Malnutrition (*n* = 208)**	
Age, M (IQR), years	102.0(3.0)	102.0(3.0)	102.0(3.0)	102.0(3.0)	0.533
Woman, %	822(82.0)	93(75.6)	545(81.2)	184(88.5)	0.008
Primary school and below, %	982(98.2)	7(5.7)	11(1.6)	2(1.0)	0.006
Living with families, %	863(86.1)	104(84.6)	565(84.2)	194(93.3)	0.004
Physical activity, %	129(12.9)	23(18.7)	93(13.9)	13(6.3)	0.002
BMI, M (IQR)	17.9(16.0-19.9)	21.8(20.3–23.8)	17.8(15.9–19.5)	17.1(16.0-18.4)	< 0.001
Hb, M (IQR), g/L	114.0(20.0)	119.0(18.0)	114.0(19.0)	112.0(21.8)	< 0.001
ALB, M (IQR), g/L	4.6(1.2)	4.8(1.2)	4.6(1.1)	4.5(1.3)	< 0.001
TC, M (IQR), mmol/L	38.5(5.1)	40.2(3.7)	38.6(4.9)	37.4(6.2)	< 0.001
Diabetes, %	96(9.6)	13(10.6)	63(9.4)	20(9.6)	0.920
Hypertension, %	757(75.5)	99(80.5)	501(74.7)	157(75.5)	0.385
Heart disease, %	41(4.1)	5(4.1)	26(3.9)	10(4.8)	0.839

^a^ All *p*-values were significant at *P* < 0.05

**Fig. 1:**
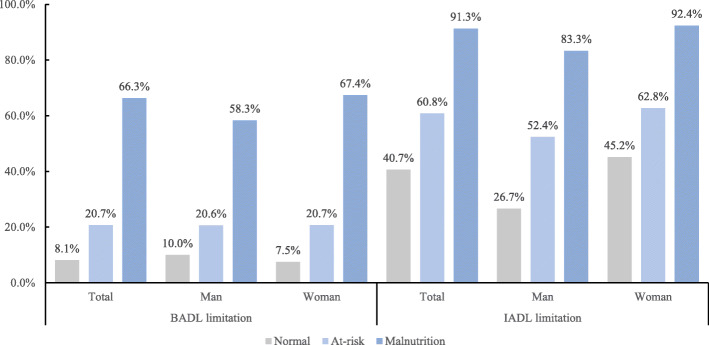
Proportion of centenarians with BADL/IADL limitation under the different nutritional status

Figure 1 shows the proportion of centenarians with BADL/IADL limitation under the different nutritional status. In the total sample, the prevalence of malnutrition was 20.8 % (208/1002), BADL limitation was 28.6 % (287/1002), and IADL limitation was 64.7 % (648/1002). The malnutrition group had a significantly higher prevalence of ADL limitation than the prevalence of other groups. There was no statistical difference between men and women with BADL limitation (*p* = 0.120), while IADL limitation did (*p* < 0.001).

**Fig. 2:**
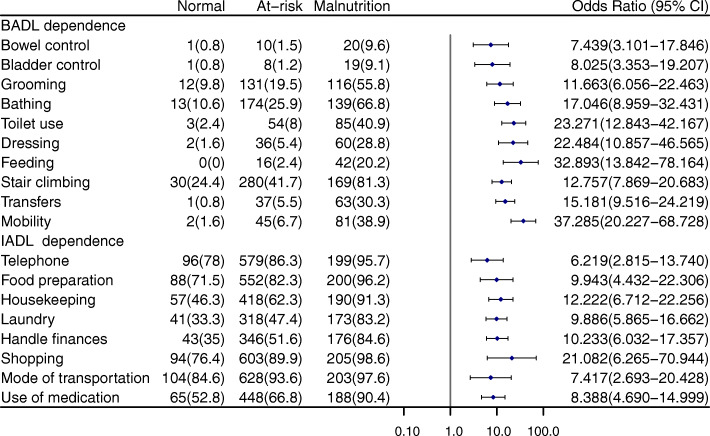
Functional status of centenarians according to nutritional status [n (%)]

Figure 2 shows the proportion of centenarians who are functionally independent under different nutritional status, and the odds ratio of malnutrition group to normal nutrition group. The proportion of centenarians with various dependencies increased with nutritional status deteriorated. In BADL, bladder control is a relatively independent behavior (28/1002); the proportion of bladder control is also lower than other behavior under different nutritional status. Stair climbing was the least independent behavior. In IADL, laundry is the most independent behavior (532/1002), and mode of transportation is the least independent behavior.

Tables 2 and 3 present the results of multivariable logistic regression analysis for the association between nutritional status and BADL/IADL. As the nutritional status deteriorated, the risk of ADL limitations gradually increased (*p* for trend < 0.001). In the total population, the OR of BADL limitation for malnutrition centenarians was 17.060 (95 % confidence interval [CI]: 8.093-35.964), and that of IADL was 11.221 (95 % CI: 5.853-21.511). After gradually adjusting the covariates, the OR was weakened but in the same direction. Similar results were found in both men and women but were more prominent in women after stratifying sex.

**Table 2:** Multivariable logistic regression analysis for the association between nutritional status and BADL limitation ^a, b^

	Nutritional status			*p* for trend
	**Normal**	**At risk**	**Malnutrition**	
Total population				
Crude model	1	2.952(1.506–5.787)	22.277(10.978–45.208)	˂0.001
Model 1	1	2.864(1.448–5.664)	20.955(10.169–43.179)	˂0.001
Model 2	1	2.388(1.190–4.792)	16.770(7.972–35.280)	˂0.001
Model 3	1	2.390(1.189–4.804)	17.060(8.093–35.964)	˂0.001
Man				
Crude model	1	2.340(0.658–8.319)	12.600(2.977–53.323)	˂0.001
Model 1	1	2.457(0.652–9.260)	12.438(2.702–57.259)	˂0.001
Model 2	1	2.201(0.538–9.004)	19.033(3.454-104.881)	˂0.001
Model 3	1	2.262(0.548–9.334)	19.658(3.497–110.510)	˂0.001
Woman				
Crude model	1	3.214(1.447–7.136)	25.390(11.074–58.213)	˂0.001
Model 1	1	3.176(1.421–7.102)	25.071(10.790-58.253)	˂0.001
Model 2	1	2.731(1.196–6.240)	19.913(8.340-47.548)	˂0.001
Model 3	1	2.765(1.207–6.336)	20.824(8.680–49.960)	˂0.001

^a^ Crude model: None adjusted; Model 1: Adjusted for age, sex, educational level, residence types, physical activity; Model 2: Model 1 plus hemoglobin, serum albumin, total cholesterol; Model 3: Model 2 plus diabetes, hypertension, heart disease

^b^ Described as OR (95 %CI)

**Table 3:** Multivariable logistic regression analysis for the association between nutritional status and IADL limitation ^a^

	Nutritional status			*p* for trend
	**Normal**	**At risk**	**Malnutrition**	
Total population				
Crude model	1	2.265(1.531–3.351)	15.411(8.436–28.153)	˂0.001
Model 1	1	2.221(1.457–3.385)	13.298(7.069–25.019)	˂0.001
Model 2	1	1.919(1.247–2.952)	10.880(5.700-20.769)	˂0.001
Model 3	1	1.908(1.236–2.945)	11.221(5.853–21.511)	˂0.001
Man				
Crude model	1	3.025(1.253–7.304)	13.750(3.585–52.740)	˂0.001
Model 1	1	2.929(1.126–7.617)	11.802(2.828–49.255)	˂0.001
Model 2	1	2.483(0.947–6.514)	9.941(2.301–42.960)	˂0.001
Model 3	1	2.629(0.958–7.214)	11.146(2.452–50.664)	˂0.001
Woman				
Crude model	1	2.046(1.313–3.188)	14.745(7.462–29.134)	˂0.001
Model 1	1	2.099(1.308–3.368)	13.986(6.856–28.534)	˂0.001
Model 2	1	1.828(1.124–2.972)	11.308(5.457–23.432)	˂0.001
Model 3	1	1.858(1.138–3.035)	12.007(5.761–25.027)	˂0.001

^a^ The adjusted covariates in models are the same as in Table 2

## Discussion

This is a study about the association between nutritional status and functional limitations among centenarians based on a large sample of the Chinese community population. Some studies have found that centenarians were facing functional limitations. A study describing centenarians in Mexico City showed that their ADL limitations proportion was 70.7 % [28]. Similarly, a study of 43 centenarians in Costa Rica showed that there were only 2 centenarians with independent BADL function and only 1 with IADL independence [15]. In developed countries, similar results were also found [29–31]. Consistent with studies on centenarians in other parts of China, the prevalence of BADL limitation and malnutrition among centenarians in Hainan is low, but IADL is high [32–34]. The reason for this difference is Lawton IADL assessed the instrumental activities of daily living, which require greater complexity of the neurophysical functions [35].

Although the results reported by each country and region were relatively consistent, there were no specific analysis of the impact of the nutritional status of centenarians on the activities. This study focuses on centenarians in the community, and the association between nutritional status and functional limitations. In this study of these extreme survivors, we found that malnutrition was significantly associated with the limitations of BADL and IADL among centenarians.

The results were consistent with previous studies showing that there are more woman centenarians than man centenarians in the same area [4, 14, 36]. But the functional limitations faced by man and woman were similar. The association between nutritional status and functional limitations is complex because it involves multiple system linkages. As the nutritional status deteriorated, the risk of ADL limitations gradually increased. There were different statistical differences between men and women in BADL/IADL, and one of the reasonable explanations is the difference in the education level. In this study, women’s education level was significantly lower than men’s education level (junior high school and above, 0.5 %: 8.9 %). It also requires further research to confirm the mechanism. Women are more affected by the impact of nutrition on activity, which suggests that we need to pay more attention to woman centenarians.

The results also extend the findings of the previous studies: the differences in the association between malnutrition and impaired ADL items for centenarians in different countries. In the study of centenarians in Sweden [4], Denmark [14], and Japan [37], bathing suffered the most damage in BADL, while in China it was “mobility” [36]. Although the highest proportion of impaired items is stair climbing in this study, the proportion of transfers is similar to centenarians in other parts of China. There may be two reasons for this difference. First, most centenarians in Hainan live in bungalows, and they rarely use stairs. Second, bed/chair transfer can be used to screen for long-term risks of functional limitations [38], which is more obvious for extreme survivor.

This study has some limitations. First, this research was a cross-sectional study that limits the ability to determine the causal relationship and reason between nutritional status and functional limitations. Second, we cannot obtain the association between nutritional status and functional limitations for centenarians who unable to participate in the physical examination. Last, compared with the current well-known cohort studies, the sample size of centenarians in CHCCS is smaller. It had an impact on the accuracy of the research.

## Conclusions

This study provides evidence that malnutrition is associated with functional limitations among centenarians in China and more pronounced among women. Although the prevalence of ADL limitations among centenarians in Hainan, China is low, the impaired activities of the older caused by malnutrition still needs attention. Strengthening the nutrition of the older is of practical significance for promoting healthy aging.

## Data Availability

The datasets used and/or analyzed during the current study are available from the corresponding author on reasonable request.

## Abbreviations

ADL: activities of daily living
BADL: basic ADL
IADL: instrumental ADLs
MNA-SF: the Mini Nutritional Assessment Short-Form
CHCCS: the China Hainan Centenarian Cohort Study
BMI: Body Mass Index
Hb: hemoglobin
ALB: serum albumin
TC: total cholesterol
M: median
IQR: interquartile range
OR: odds ratios
CI: confidence interval

## References

[1] Hall KS, Cohen HJ, Pieper CF, Fillenbaum GG, Kraus WE, Huffman KM (2017). Physical Performance Across the Adult Life Span: Correlates With Age and Physical Activity. J Gerontol A Biol Sci Med Sci.

[2] Brownie S (2006). Why are elderly individuals at risk of nutritional deficiency?. Int J Nurs Pract.

[3] Fauth EB, Gerstorf D, Ram N, Malmberg B (2014). Comparing changes in late-life depressive symptoms across aging, disablement, and mortality processes. Dev Psychol.

[4] Rasmussen SH, Thinggaard M, Hojgaard MB, Jeune B, Christensen K, Andersen-Ranberg K (2018). Improvement in Activities of Daily Living Among Danish Centenarians?-A Comparative Study of Two Centenarian Cohorts Born 20 Years Apart. J Gerontol A Biol Sci Med Sci.

[5] Naseer M, Forssell H, Fagerstrom C (2016). Malnutrition, functional ability and mortality among older people aged 60 years: a 7-year longitudinal study. Eur J Clin Nutr.

[6] Valentini A, Federici M, Cianfarani MA, Tarantino U, Bertoli A (2018). Frailty and nutritional status in older people: the Mini Nutritional Assessment as a screening tool for the identification of frail subjects. Clin Interv Aging.

[7] Ahmed N, Choe Y, Mustad VA, Chakraborty S, Goates S, Luo M (2018). Impact of malnutrition on survival and healthcare utilization in Medicare beneficiaries with diabetes: a retrospective cohort analysis. BMJ Open Diabetes Res Care.

[8] Maseda A, Diego-Diez C, Lorenzo-Lopez L, Lopez-Lopez R, Regueiro-Folgueira L, Millan-Calenti JC (2018). Quality of life, functional impairment and social factors as determinants of nutritional status in older adults: The VERISAUDE study. Clin Nutr.

[9] Kuzuya M (2021). Nutritional status related to poor health outcomes in older people: Which is better, obese or lean?. Geriatr Gerontol Int.

[10] Zhang X, Edwards BJ (2019). Malnutrition in Older Adults with Cancer. Curr Oncol Rep.

[11] Williams GR, Rier HN, McDonald A, Shachar SS (2019). Sarcopenia & aging in cancer. J Geriatr Oncol.

[12] Hairi NN, Cumming RG, Naganathan V, Handelsman DJ, Le Couteur DG, Creasey H (2010). Loss of muscle strength, mass (sarcopenia), and quality (specific force) and its relationship with functional limitation and physical disability: the Concord Health and Ageing in Men Project. J Am Geriatr Soc.

[13] Nieddu A, Vindas L, Errigo A, Vindas J, Pes GM, Dore MP (2020). Dietary Habits, Anthropometric Features and Daily Performance in Two Independent Long-Lived Populations from Nicoya peninsula (Costa Rica) and Ogliastra (Sardinia). Nutrients.

[14] Vetrano DL, Grande G, Marengoni A, Calderon-Larranaga A, Rizzuto D (2021). Health Trajectories in Swedish Centenarians. J Gerontol A Biol Sci Med Sci.

[15] Madrigal-Leer F, Martìnez-Montandòn A, Solìs-Umaña M, Helo-Guzmàn F, Alfaro-Salas K, Barrientos-Calvo I (2020). Clinical, functional, mental and social profile of the Nicoya Peninsula centenarians, Costa Rica, 2017. Aging Clin Exp Res.

[16] Pérez-Ros P, Vila-Candel R, López-Hernández L, Martínez-Arnau FM. Nutritional Status and Risk Factors for Frailty in Community-Dwelling Older People: A Cross-Sectional Study. Nutrients. 2020;12(4):1041. 10.3390/nu12041041 PMC7231056 32290060

[17] Sebastiani P, Federico A, Morris M, Gurinovich A, Tanaka T, Chandler KB (2021). Protein signatures of centenarians and their offspring suggest centenarians age slower than other humans. Aging Cell.

[18] He Y, Zhao Y, Yao Y, Yang S, Li J, Liu M, et al. Cohort Profile: The China Hainan Centenarian Cohort Study (CHCCS). Int J Epidemiol. 2018; 47(3):694-5 h. 10.1093/ije/dyy017 29506028

[19] Mahoney FI, Barthel DW (1965). Functional Evaluation: The Barthel Index. Md State Med J.

[20] Katz S (1983). Assessing self-maintenance: activities of daily living, mobility, and instrumental activities of daily living. J Am Geriatr Soc.

[21] Ng TP, Niti M, Chiam PC, Kua EH (2006). Physical and cognitive domains of the Instrumental Activities of Daily Living: validation in a multiethnic population of Asian older adults. J Gerontol A Biol Sci Med Sci.

[22] Leung SO, Chan CC, Shah S (2007). Development of a Chinese version of the Modified Barthel Index– validity and reliability. Clin Rehabil.

[23] Jopp DS, Park MK, Lehrfeld J, Paggi ME (2016). Physical, cognitive, social and mental health in near-centenarians and centenarians living in New York City: findings from the Fordham Centenarian Study. BMC Geriatr.

[24] Mlinac ME, Feng MC (2016). Assessment of Activities of Daily Living, Self-Care, and Independence. Arch Clin Neuropsychol.

[25] Lawton MP, Brody EM (1969). Assessment of older people: self-maintaining and instrumental activities of daily living. Gerontologist.

[26] Chivite D, Formiga F, Corbella X, Conde-Martel A, Aramburu O, Carrera M (2018). Basal functional status predicts one-year mortality after a heart failure hospitalization in elderly patients - The RICA prospective study. Int J Cardiol.

[27] Kaiser MJ, Bauer JM, Ramsch C, Uter W, Guigoz Y, Cederholm T (2009). Validation of the Mini Nutritional Assessment short-form (MNA-SF): a practical tool for identification of nutritional status. J Nutr Health Aging..

[28] Pedro VC, Arturo RH, Alejandro PM, Oscar RC (2017). Sociodemographic and Clinical Characteristics of Centenarians in Mexico City. Biomed Res Int..

[29] Martin P, Gondo Y, Arai Y, Ishioka Y, Woodard JL, Poon LW (2018). Physical, sensory, and cognitive functioning among centenarians: a comparison between the Tokyo and Georgia centenarian studies. Qual Life Res.

[30] Herr M, Jeune B, Fors S, Andersen-Ranberg K, Ankri J, Arai Y (2018). Frailty and Associated Factors among Centenarians in the 5-COOP Countries. Gerontology.

[31] Daniels LB, Antonini P, Marino R, Rizzo M, Navarin S, Lucibello SG (2020). Cardiovascular health of nonagenarians in southern Italy: a cross-sectional, home-based pilot study of longevity. J Cardiovasc Med (Hagerstown).

[32] Liang Y, Welmer AK, Wang R, Song A, Fratiglioni L, Qiu C (2017). Trends in Incidence of Disability in Activities of Daily Living in Chinese Older Adults: 1993–2006. J Am Geriatr Soc.

[33] Wu T, Lu L, Luo L, Guo Y, Ying L, Tao Q (2017). Factors Associated with Activities of Daily Life Disability among Centenarians in Rural Chongqing, China: A Cross-Sectional Study. Int J Environ Res Public Health.

[34] Xu X, Yang L, Miao X, Hu X (2020). An investigation and analysis of the activities of daily living of older adults living at home in Ningxia Hui Autonomous Region of China: a cross-sectional study. BMC Geriatr.

[35] de Oliveira LFS, Wanderley RL, de Medeiros MMD, de Figueredo OMC, Pinheiro MA, Rodrigues Garcia RCM (2021). Health-related quality of life of institutionalized older adults: Influence of physical, nutritional and self-perceived health status. Arch Gerontol Geriatr.

[36] Huang Z, Chen Y, Zhou W, Li X, Qin Q, Fei Y (2020). Analyzing functional status and its correlates in Chinese centenarians: A cross-sectional study. Nurs Health Sci.

[37] Martin P, Gondo Y, Arai Y, Ishioka Y, Woodard J, Poon L (2018). Physical, sensory, and cognitive functioning among centenarians: a comparison between the Tokyo and Georgia centenarian studies. Qual Life Res.

[38] Ferreira MS, de Melo Franco FG, Rodrigues PS, da Silva de Poli Correa VM, Akopian STG, Cucato GG (2019). Impaired chair-to-bed transfer ability leads to longer hospital stays among elderly patients. BMC Geriatr.

